# Transcriptome-Wide Study of mRNAs and lncRNAs Modified by m^6^A RNA Methylation in the *Longissimus Dorsi* Muscle Development of Cattle-Yak

**DOI:** 10.3390/cells11223654

**Published:** 2022-11-17

**Authors:** Chun Huang, Rongfeng Dai, Guangyao Meng, Renqing Dingkao, Xingdong Wang, Wenwen Ren, Xiaoming Ma, Xiaoyun Wu, Min Chu, Yongfu La, Pengjia Bao, Xian Guo, Jie Pei, Ping Yan, Chunnian Liang

**Affiliations:** 1 Key Laboratory of Yak Breeding Engineering Gansu Province, Lanzhou Institute of Husbandry and Pharmaceutical Science, Chinese Academy of Agricultural Sciences, Lanzhou 730050, China; 2 Key Laboratory of Animal Genetics and Breeding on Tibetan Plateau, Ministry of Agriculture and Rural Affairs, Lanzhou 730050, China; 3 Animal Husbandry Station of Gannan Tibetan Autonomous Prefecture, Gannan 747000, China

**Keywords:** cattle-yak, N6-methyladenosine, mRNA and lncRNA, muscle development

## Abstract

Cattle-yak is a hybrid F_1_ generation of cattle and yak, which has a history of more than 3000 years and has shown better production performance and higher economic benefits than those of yaks. However, up to now, there has been no study on the transcriptome-wide m^6^A methylation profile of bovine skeletal muscle and its potential biological function during muscle development. Here, we observed significant changes in the expression levels of muscle-related marker genes and methylation-related enzymes during the development of cattle-yak, and the overall m^6^A content in the *Longissimus dorsi* muscle of 18-month-old cattle-yak decreased significantly. A total of 36,602 peaks, 11,223 genes and 8388 lncRNAs were identified in the two groups, including 2989 differential peaks (427 up-regulated peaks and 2562 down-regulated peaks), 1457 differentially expressed genes (833 up-regulated genes and 624 down-regulated genes) and 857 differentially expressed lncRNAs (293 up-regulated lncRNAs and 564 down-regulated lncRNAs). GO and KEGG analysis revealed that they were significantly enriched in some muscle-related pathways (Wnt signaling pathway and MAPK signaling pathway) and high-altitude adaptation-related pathway (HIF-1 signaling pathway). Moreover, m^6^A abundance was positively correlated with gene expression levels, while it was negatively correlated with lncRNA expression levels. This indicates that m^6^A modification played an important role in the *Longissimus dorsi* muscle development of cattle-yak; however, the regulation mechanism of m^6^A-modified mRNA and lncRNA may be different. This study was the first report of transcriptome-wide m^6^A-modified mRNAs and lncRNAs atlas in the *Longissimus dorsi* muscle development of cattle-yak, one which will provide new perspectives for genetic improvement in bovines.

## 1. Introduction

Cattle-yak, as the hybrid F_1_ generation of cattle (*Bos taurus*) and yak (*Bos grunniens*), has a history of more than 3000 years and gradually forms a new native breed after long-term domestication. In high-altitude hypoxic areas such as the Qinghai-Tibet Plateau, the production performance and meat quality of the yaks have been unable to meet human needs, while cattle-yaks have shown obvious hybrid vigor. In previous studies, the production performance of cattle-yaks at all growth stages was significantly higher than that of yaks [1]. At the same time, the meat quality of cattle-yaks has higher protein and lower fat content, which can satisfy the requirements of human health and high-quality diet better than yaks [2,3]. Given this, it is of great significance for genetic breeding improvement to clarify the muscle development mechanism of cattle-yak. Skeletal muscle is known as an extremely important tissue; it not only acts a crucial role in regulating muscle quality in animals, but also plays an irreplaceable role in bodily health [4,5]. The reports revealed that the muscle cell proliferation and differentiation were precisely controlled by myogenic genes and transcription factors through strict time-series expression [6,7,8]. It is well known that the myogenic regulatory factors (*MRFs*) family, including myogenic determining factor (*MyoD*), myogenic factor 5 (*Myf5*), myogenic protein (*MyogG*) and *MRF4* take the decisive regulatory effects in skeletal muscle development [9]. These genes mainly take part in the skeletal muscle growth process, including myoblast differentiation, inducing terminal differentiation, promoting fusion into multinucleated myotubes and muscle fiber maintenance [10,11,12]. In addition, long non-coding RNA (lncRNA) occupied most of the transcripts in the mammalian genome and also worked in a specific place in skeletal muscle development. The first identified lncRNA in mammalian, lncRNA-*H19*, was able to inhibit the proliferation and differentiation of C2C12 myoblasts line by suppressing the expression level of *Smad* family members [13]. Likewise, LncRNA-*FKBP1C* could control muscle fiber type by affecting the stability of *MYH1B* [14]. More and more lncRNAs have been reported to regulate the skeletal muscle maintenance or development, such as linc-*MD1* [15], lncR-*lrm* [16] and lnc-*Myod* [17,18].

Besides the important role of a series of transcription factors in skeletal muscle development, the epigenetic modifications such as methylation are also critical for muscle growth [19,20]. Nevertheless, the genetic improvement of yak, especially the heterosis of its hybrid offspring, cattle-yak, has always focused on the phenotypic research and DNA level exploration, and rarely investigated how to promote the breeding process from the perspective of RNA modification. N6-methyladenosine (m^6^A) is ubiquitous in mRNA modification in eukaryotes [21,22,23,24,25], and most RNA modifications were RNA methylation [26]. m^6^A modification is a dynamic and reversible process that is mediated by multiple binding proteins to regulate mRNA splicing, expression, decay and translation processes [27,28,29], including methyltransferases (*METTL1/3/14* and *WTAP*, etc.) [30,31], demethylases (*ALKBH5* and *FTO*, etc.) [32,33] and reader (*YTHDF1/2/3* and *YTHDC*, etc.) [34]. *FTO* could promote adipocyte proliferation by inhibiting the *Wnt*/β-catenin signaling pathway in porcine adipocytes, thereby promoting adipogenesis [35]. However, the expression level of *FTO* was negatively correlated with fat deposition in skeletal muscle, which in turn affected skeletal muscle development [36]. *METTL3*-mediated m^6^A methylation was identified as having many features in skeletal muscle, such as affecting muscle regeneration and myocyte state transition [37,38]. Although m^6^A-modified lncRNAs have gradually attracted the attention of researchers, the distribution characteristic and biological function of m^6^A in ncRNAs and regulatory elements are still unclear. Wang et al. described a m^6^A methylation profile of lncRNAs in porcine oxidized and glycolytic skeletal muscle, identifying seven m^6^A-modified lncRNAs that may play key roles in muscle fiber conversion [39]. In mouse C2C12 muscle cell lines, the methylation level of lncRNAs was positively correlated with the abundance of lncRNAs transcripts, and the m^6^A-modified lncRNA map was plotted at different growth periods [40]. These studies have demonstrated that m^6^A modification on mRNA or lncRNA was of great importance to animal myogenesis. In contrast, up to now, the m^6^A modification on mRNA and lncRNA profiles of skeletal muscle in cattle-yak have still not been reported. More importantly, m^6^A-modified lncRNAs have never been studied in skeletal muscle development of large domestic animals.

Here, we identified muscle-related marker genes in the *Longissimus dorsi* muscle of cattle-yak at different developmental stages, then obtained the first transcriptome-wide m^6^A methylation profile of cattle-yak skeletal muscle development by MeRIP-seq and RNA-seq techniques and clarified the characteristics of m^6^A modification. Moreover, we reassembled the transcripts and analyzed the characteristics of m^6^A-modified lncRNAs in skeletal muscle development of bovines for the first time. This work will complement the study of m^6^A methylation in plateau livestock, and provide new ideas for exploring the role of m^6^A in skeletal muscle and the distribution and biological function of m^6^A in ncRNAs.

## 2. Results

### 2.1. Global m^6^A Modification Patterns in the Longissimus Muscle of Cattle-Yaks from Different Ages

**Figure 1:**
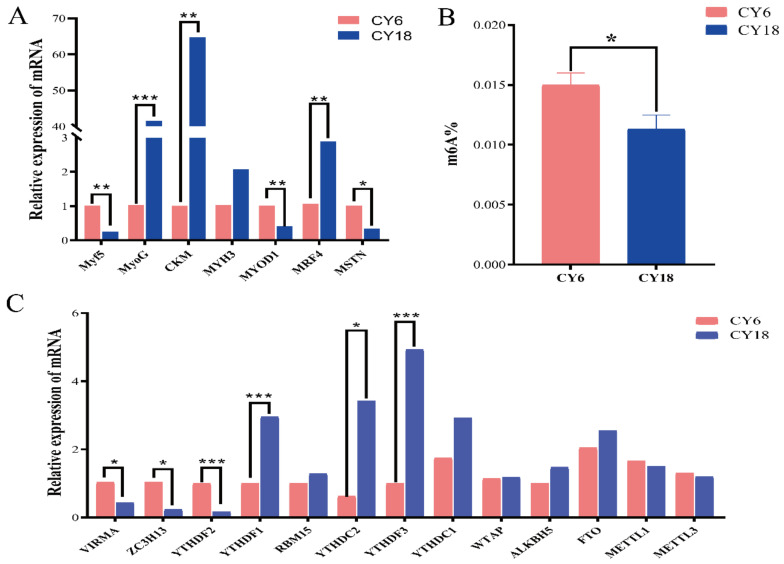
The analysis of muscle-related marker genes and the detection of m^6^A level at different development stages of cattle-yaks: (**A**) relative expression of muscle-related marker genes between six-month-old cattle-yaks (CY6) and eighteen-month-old cattle-yaks (CY18); (**B**) overall m^6^A content between CY6 and CY18; (**C**) the identification of expression levels of methylation-related genes. “***” *p* < 0.001, “**” *p* < 0.01, “*” *p* < 0.05.

The *MYOD1*, *MyoG*, *MYH3*, *MRF4* and *CKM* genes are considered as marker genes for differentiated myoblasts and fused myotubes, which are significantly correlated [41,42]. Therefore, we evaluated the relative mRNA expression levels of these genes to verify whether the weight gain of cattle-yak was consistent with the related function of these marker genes (Figure 1A). Obviously, the expression levels of the *MyoG*, *CKM*, *MRF4* genes were significantly increased in cattle-yaks at eighteen months in age. Moreover, the *MSTN* gene, as the inhibitor of skeletal muscle development [43], was significantly decreased with the increase of cattle-yak age. Meanwhile, there were significant decreases in the expression level of the *Myf5* and *MYOD1* genes of eighteen-month-old cattle-yaks.

Since m^6^A modification is instrumental in muscle development and growth, we quantified the global m^6^A modifications using colorimetry and the expression levels in six-month-old and eighteen-month-old cattle-yaks. As shown in Figure 1B, compared with the cattle-yaks of six months in age, the content of m^6^A in the cattle-yaks of eighteen months in age was significantly lower. Subsequently, the results of qRT-PCR showed that the expression levels of methyltransferases (*METTL1/3* and *WTAP* genes) and demethylases (*FTO* and *ALKBH5*) were not significantly changed with age (Figure 1C). However, the methyltransferases *ZC3H13* and *VIRMA* genes were significantly down-regulated in the eighteen-month-old cattle-yaks. Conversely, the methylation reader proteins with *YTH* domain including *YTHDF1/3* and *YTHDC2* were significantly increased in the *Longissimus dorsi* muscle of eighteen-month-old cattle-yaks. The above results indicated that m^6^A modifications may play a vital potential regulatory role in muscle development of cattle-yak.

### 2.2. Transcriptome-Wide m^6^A- seq Reveals Global m^6^A Modification Patterns from Longissimus Dorsi Muscle of Cattle-Yaks

The transcriptome m^6^A-seq and RNA-seq (input) were performed to investigate the role of m^6^A modifications in muscle development of cattle-yak. Three biologically repeated samples in each group were sequenced to ensure the repeatability of this work. A total of 12 libraries were constructed, including input and MeRIP results. After a series of rigorous screenings, we reserved a mean of 88.34 M high-quality clean reads per sample. The valid data were mapped to the reference genome by Hisat2, and the proportion of mapped reads ranged from 87.44 to 92.34% (Supplementary Table S2).

**Figure 2:**
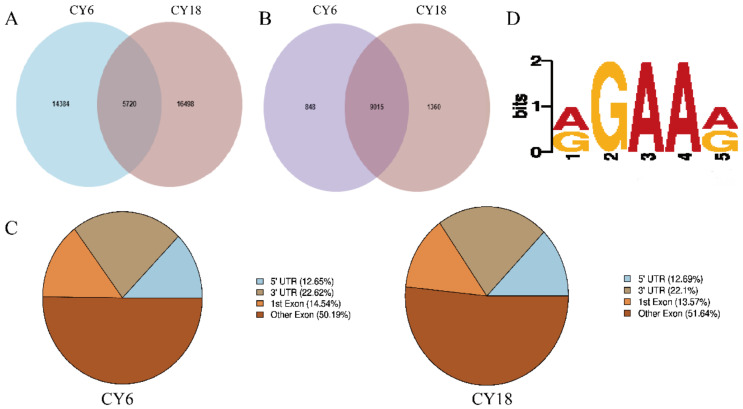
Transcriptome-wide m^6^A-seq and m^6^A peak analysis: (**A**) the common and specific m^6^A peaks in two groups; (**B**) the m^6^A peaks for genes in two groups of Venn diagrams; (**C**) pie chart for the peak in gene functional element region annotation; (**D**) sequence logo representing the consensus motif identified by DREME.

Peak calling was performed by MeTDiff software, and we detected 20,104 and 22,218 peaks in Group eighteen-month-old cattle-yaks (CY18) and six-month-old cattle-yaks (CY6), respectively. The peaks in the two groups represented transcripts of 9863 genes and 10,375 genes. Among them, there were 14,384 CY6-unique peaks and 16,498 CY18-unique peaks, respectively (Figure 2A,B). Interestingly, the results showed that the m^6^A peaks per gene in cattle-yak skeletal muscle (~3.0) were generally higher than those in bovine skeletal myoblasts and myotubes (~1.60 m^6^A peaks per m^6^A transcript) [44], human HepG2 (∼1.7 m^6^A peaks per gene) [45] and pig adipose tissue (∼1.3 m^6^A peaks per gene) [46]. It can be seen from Figure 2C that most of the peaks were concentrated in the exon region, followed by the 3’UTR and 5’UTR regions, and the enriched results were consistent with previous reports of yaks [47,48]. Next, the significant peaks were analyzed to determine whether m^6^A peaks contained the m^6^A methyltransferase-combined consensus motifs. Consistent with previous reports, the results showed that the m^6^A peaks were typically present in RGAAR (R = A or G) motif [49], which enhances credibility of the m^6^A peaks (Figure 2D).

### 2.3. Analysis of Different Methylated Peaks (DMPs) in Different Development Stages

**Figure 3:**
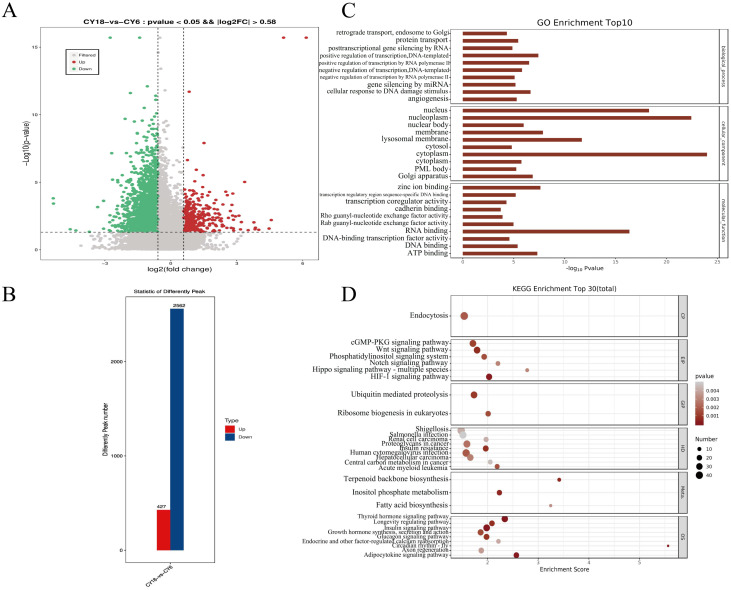
Analysis of the DMPs in cattle-yaks of different development stages: (**A**) volcano plots showing the DMPs between two groups; (**B**) number of up- and down-regulated DMPs; (**C**) GO analysis of DMPs; (**D**) the top 30 significant enriched pathways of DMPs.

To further understand the potential function of m^6^A peaks’ represented genes, we performed the comparison for the abundance of m^6^A peaks between two groups. The 2989 differential peaks were scanned compared to the cattle-yaks of six months old, which contained 427 up-regulated peaks and 2562 down-regulated peaks (*p* <0.05, Fold Change >1.5) (Supplementary Table S3, Figure 3A,B). Moreover, GO and KEGG pathway enrichment analysis were performed to analyze the potential function of m^6^A peak-related genes. According to the GO analysis results, the differential methylated genes were enriched significantly in the terms of molecular function (MF), cellular component (CC) and biological process (BP), such as positive regulation of transcription by RNA polymerase II, protein transport, ATP binding and DNA-binding transcription factor activity et al. (Figure 3C). KEGG analysis showed DMPs enriched in some signal pathways related to the disease and high-altitude adaptation, similarly, many signaling pathways related to muscle growth were found to be enriched, including Hippo signaling pathway, Wnt signaling pathway, Notch signaling pathway, and Growth hormone synthesis, secretion and action (Figure 3D).

### 2.4. Analysis of Differentially Expressed Genes (DEGs) between Two Groups by RNA-seq

**Figure 4:**
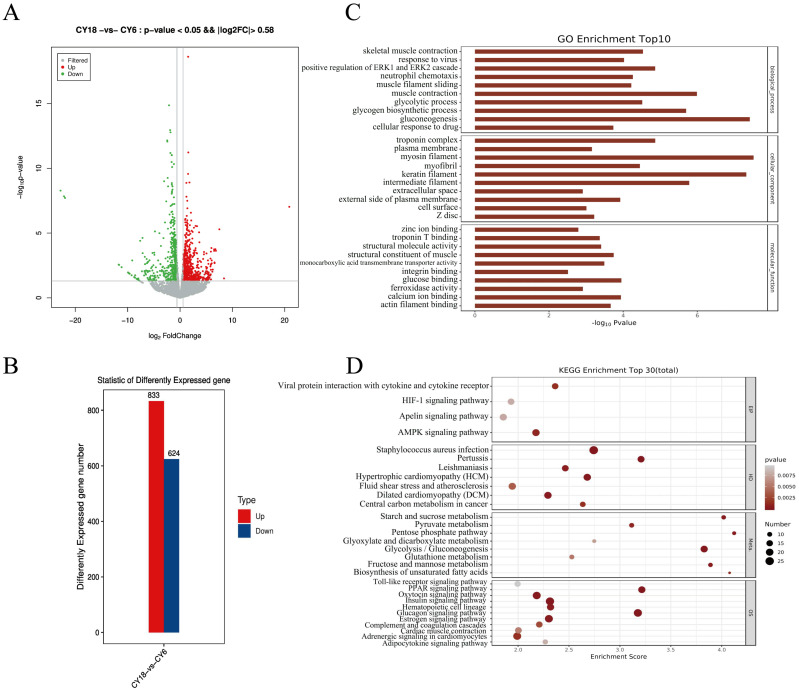
RNA-seq analysis of DEGs between two groups: (**A**) volcano plots representing the DEGs; (**B**) number of up- and down-regulated DEGs; (**C**) GO analysis of DEGs; (**D**) KEGG pathway analysis of DEGs.

All samples were analyzed by RNA-seq in view of m^6^A abundance on mRNA levels [24,47,50]. As shown in Figure 4, there were considerable differences of global mRNA expression between CY6 and CY18. We found a total of 1457 differentially expressed genes (DEGs) between two groups, including 833 up-regulated genes and 624 down-regulated genes compared to group CY6 (Supplementary Table S4, Figure 4A,B). The heatmap construction and clustering analysis were performed using unsupervised hierarchical clustering analysis to further explore the global expression changes and potential roles of DEGs (Supplementary Figure S1). Next, we further analyzed the potential function of DEGs using GO and KEGG pathway analysis. GO analysis revealed that DEGs were enriched in muscle-related terms including skeletal muscle contraction, actin filament binding and structural constituent of muscle (Figure 4C). By means of the KEGG analysis, it was uncovered that DEGs were significantly enriched in AMPK signaling pathway, HIF-1 signaling pathway and some metabolism-related and muscle-related pathways, including the PPAR signaling pathway, Fructose and mannose metabolism and Biosynthesis of unsaturated fatty acids pathways (Figure 4D).

### 2.5. Integrated Analysis of m^6^A-seq and RNA-seq Data between CY6 and CY18

**Figure 5:**
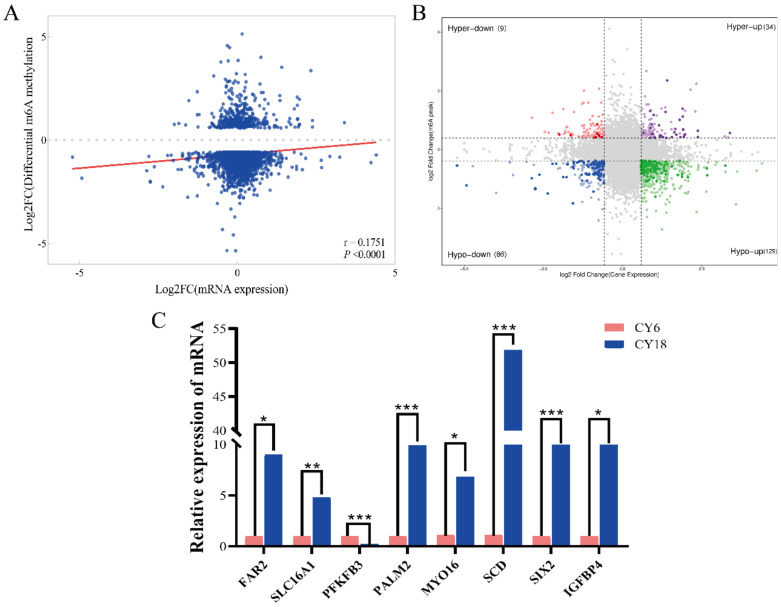
Integrated analysis of DEPs and DEGs: (**A**) positive correlation between m^6^A methylation and mRNA expression level (r = 0.1751, *p* < 0.001); (**B**) distribution of genes with a significant change in m^6^A methylation level and gene expression; (**C**) the results of qRT-PCR for verifying the RNA-seq data. “***” *p* < 0.001, “**” *p* < 0.01, “*” *p* < 0.05.

To further amplify and analyze the potential role of m^6^A modification on gene expression, the conjoint analysis of MeRIP-seq and RNA-seq data were conducted. Interestingly, there was a positive correlation between DMPs and DEGs in the developmental stages of skeletal muscles based on Pearson Correlation Analysis (Figure 5A). Four types were identified in conformity with the change of expression level, namely hyper-up (hyper-methylated and mRNA up), hyper-down (hyper-methylated and mRNA down), hypo-up (hypo-methylated and mRNA up) and hypo-down (hypo-methylated and mRNA down), respectively. These types contained 34, 9, 129 and 86 genes, respectively (Figure 5B, Supplementary Table S5). Finally, we selected randomly eight genes to verify the accuracy of RNA-seq data using qRT-PCR, the results showed the overall change trend as same as RNA-seq data, indicating that the RNA-seq data were reliable (Figure 5C).

### 2.6. Comprehensive Analysis of Potential Peaks in lncRNAs in the Longissimus Dorsi Muscle of Cattle-Yaks

**Figure 6:**
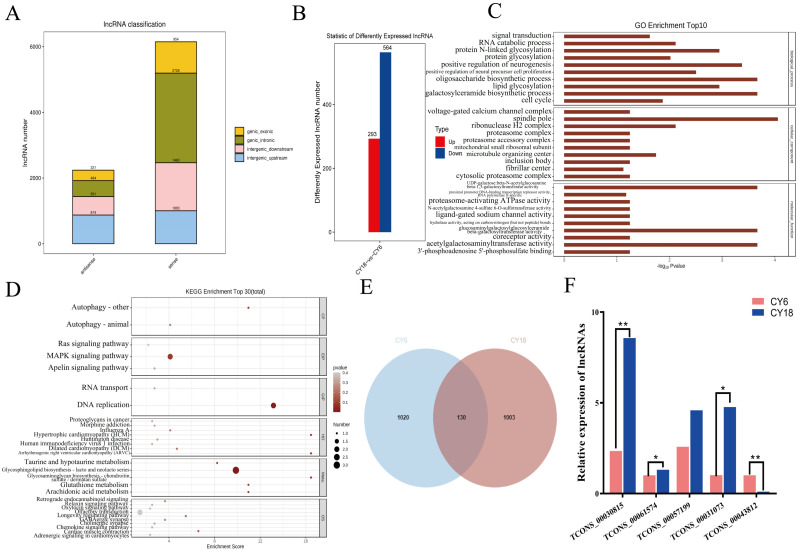
Identification and characterization of lncRNAs identified in the *Longissimus dorsi* muscle of cattle-yaks at different development stages: (**A**) classification of the identified lncRNAs in group CY6 and CY18; (**B**) number of up- and down-regulated differentially expressed lncRNAs (DELs); (**C**) GO analysis of DELs; (**D**) KEGG pathway analysis of DELs; (**E**) the common and specific m^6^A peaks on the lncRNAs in two groups; (F) the differentially expressed lncRNAs were testified using qRT-PCR. “**” *p* < 0.01, “*” *p* < 0.05.

The transcripts were reassembled to perform the lncRNA prediction and potential peaks analysis on lncRNAs using String Tie software. After a rigorous screening process, 8388 lncRNAs were identified. As shown in Figure 6A, about 73.30 % of the lncRNAs were sense lncRNAs. According to the results of FC > 1.5 and *p* < 0.05, a total of 857 lncRNAs were finally identified as differentially expressed lncRNAs (DELs). Moreover, compared with the cattle-yaks of group CY6, we found 293 up-regulated lncRNAs and 564 down-regulated lncRNAs (Figure 6B). To further explore the potential functions of the DELs, GO and KEGG pathway analysis were performed. The GO results showed that the DELs were significantly enriched in cell cycle, protein glycosylation and signal transduction terms (Figure 6C). According to the KEGG analysis, there were some disease-related and muscle-related pathways to be significantly enriched in, such as hypertrophic cardiomyopathy pathway, MAPK signaling pathway and cardiac muscle contraction (Figure 6D).

The location information of the identified was compared with the known lncRNA annotation information in NCBI and Ensembl databases. If there was overlap between the two, it was considered that the peak may be in lncRNA. We detected 1150 and 1133 peaks on the lncRNAs in the CY6 and CY18, respectively (Figure 6E). And there were 130 shared peaks between the two groups. Conjoint analysis of m^6^A and lncRNAs data was performed to identify the potential role of m^6^A on lncRNAs. There were 8 lncRNAs with a marked change in both RNA expressions and m^6^A methylation levels between group CY6 and CY18 (Table 1). Moreover, the five lncRNAs were randomly selected to test the expression levels, the results showed that the sequencing data was accurate and credible (Figure 6F).

## 3. Discussion

Skeletal muscle development is a continuous and complex process that serves crucial roles in the body health [51]. Skeletal muscle, as the most abundant tissue in domesticated animals, is closely related to meat production and is one of the most important economic traits, especially in animal husbandry [52,53]. Currently, more than 150 types of post-transcriptional modifications have been discovered in all living organisms, of which more than 60 % are RNA methylation [26,54]. Meanwhile, N^6^-methyladenosine (m^6^A) is considered the most common internal mRNA and lncRNA modification in eukaryotes [23,24,25,55,56]. However, the molecular breeding of yak and cattle-yak has mainly been concerned with the study of certain functional genes, and has rarely further explored the functional mechanism from the perspective of RNA. In the meantime, m^6^A methylation has been widely performed to investigate cattle-yak infertility, but there is still no report on RNA methylation related to the cattle-yak growth and development. In other words, the small amount of research on RNA methylation is insufficient to reveal the mechanism of muscle development in cattle-yak even if cattle-yaks have obvious heterosis in production performance, which has also produced the result that the m^6^A methylation mechanisms that contribute to the growth heterosis of cattle-yaks remain largely unknown. For this, we performed MeRIP-seq technology for the first time to establish a comprehensive transcriptome-wide atlas of m^6^A modifications in muscle growth and development of cattle-yak to analyze the potential functions of m^6^A modification in the muscle growth advantage of cattle-yak.

In previous studies, the production performance of cattle-yaks was significantly higher than that of yaks at two developmental stages, hence we selected the cattle-yaks aged six months and eighteen months to explore the transcriptome-wide m6A modifications profile [1]. First, we analyzed the marker genes associated with myoblast proliferation and regulation of skeletal muscle development in conjunction with the changes in production performance using qRT-PCR technology. The sufficient evidence indicated that the *MYOG*, *MYH3* (myosin heavy chain 3), *MRF4* (myogenic regulatory factor 4) and *CKM* (creatine kinase) genes are the marker genes of myoblast differentiation [41,42]. The expression of the genes that positively regulate muscle growth was significantly increased in the cattle-yaks aged eighteen months. In contrast, *MSTN*, as an inhibitor of skeletal muscle development, showed a significant decrease with age. These results suggested that the muscle development of eighteen-month-old cattle-yak may be faster than that of six-month-old cattle-yak. *MyoD1* and *Myf5*, two crucial genes in the myogenic regulatory factor *MRFs* family, exert their effectiveness in the primary stage of muscle development, especially myoblast proliferation [57]. It is noteworthy that their expression levels in this study were opposite to those of *MyoG* and *MRF4* genes, which play an important role in the fusion and differentiation of myoblasts in the secondary stage of muscle development, and their expression levels were significantly reduced with age. It indicated that the patterns of regulating skeletal muscle growth in different growth stages of cattle-yaks were different, and the types and function of myoblasts were also different. *MyoD1* and *Myf5* genes may act as a key part to the early stage of myogenesis and development, however, the *MyoG* and *MRF4* genes may make a greater difference to promote skeletal muscle development of cattle-yaks with age. The above results will help us to more accurately improve the production performance of cattle-yak in different periods for molecular breeding.

The transcriptome-wide m^6^A profile of the *Longissimus dorsi* muscle of cattle-yaks contained a total of 36,602 peaks and 11,223 genes. On average, each m^6^A-modified gene contained more than three m^6^A peaks, indicating that m^6^A modification in cattle-yak may have a wider and more critical impact than other animals. The studies have reported that the higher levels of m^6^A methylation were needed by tissues with greater development capability to adapt to faster growth [46], suggesting that cattle-yaks may inherit the special energy metabolism mechanism of yaks and still show strong adaptability and better production performance in high-altitude hypoxic environments. Notably, the overall level of m^6^A methylation showed a significant decreasing trend with age. Consistently, m^6^A demethylases *ALKBH5* and *FTO* were more highly expressed in eighteen-month-old muscle samples than in six-month-old muscle samples, whereas the expression of m^6^A methyltransferase *METTL3* and *METTL1* were lower in group CY18 than in group CY6. And we speculated that this may be the reason for the overall decrease of m^6^A content. Moreover, it was consistent with the skeletal muscle in pigs that the overall m^6^A levels were negatively correlated with fat deposition [46]. Therefore, it was reasonable to speculate that the decrease of the overall level of m^6^A methylation during growth and development promoted the fat deposition of cattle-yaks to improve the ability to adapt to harsh environments, which was also consistent with the above conclusions. In the *Longissimus dorsi* muscle of cattle-yak, m^6^A modifications were mainly distributed in the CDS region, mRNA 5’UTR and 3’UTR, which is similar to other mammals (humans, mice, cattle, etc.) [45,58,59]. This result also showed that the overall distribution of m^6^A modification sites is similar in mammals. Nevertheless, the distribution statistics were different from those of poultry [60,61], which also provided evidence for the differential diversity of m^6^A methylation distribution. Meanwhile, m^6^A peaks were more enriched in the mRNA 3′UTR than in the mRNA 5′UTR, and the result was obviously different from other mammals [62,63]. Previous studies have shown the m^6^A modifications enriched in the mRNA 3′UTR may be linked with many functions, including mRNA stability and signaling transport, and may also be able to regulate protein translation by recruiting specific factors for RNA transport or protein synthesis [24,27,64], which may also lead to a positive correlation between m^6^A methylation and transcription levels in the *Longissimus dorsi* muscle of cattle-yak. There was no significant alteration in the mRNA 3′UTR and 5′UTR at different growth stages, showing that the higher m^6^A signal located at 3′UTR may exert an enormous function on promoting the growth and development of cattle-yak. The consensus motif “RGAAR” sequence was identified in m^6^A peaks of the *Longissimus dorsi* muscle, and it was consistent with the findings in mouse heart [49]. Interestingly, this was distinct from the results observed in most plants and mammals, therefore, it may be speculated that RNA adenosine methylation may be variable in different species, even if it is relatively conservative. There was clear-cut evidence that *YTHDF3* may partially regulate cell differentiation by cooperating with *YTHDF1* [65], and it is consistent with our results that the expression levels of the *TYHDF3* and *YTHDF1* genes were positively correlated. The *YTHDC2* gene also mediates the m^6^A binding, and is essential for embryonic differentiation. Knockout of *YTHDC2* will hinder the development of mouse germ cells and lead to infertility [66,67]. In this work, the expression level of *YTHDC2* gene showed a significant increase at the age of eighteen months, indicating that m^6^A modification not only affected mRNA expression, but also promoted mRNA translation during the growth and development of cattle-yaks. On the other hand, the marker genes (*MyoG*, *MYH3*, *MRF4* and *CKM*)-related myoblast differentiation was significantly increased in the *Longissimus* muscle of eighteen-month-old cattle-yaks, while the overall m^6^A abundance showed the obvious descent trends, which was the same as the characteristics of m^6^A modifications in bovine skeletal muscle myoblast [44]. Therefore, we reasonably speculated that the demethylases (*YTHDF1/3* and *YTHDC2*) may be able to recognize the m^6^A sites in the mRNA 3’UTR of these marker genes and promote their mRNA translation or stabilization, that is, m^6^A may accelerate muscle development by mediating the m^6^A levels of these genes. Certainly, this conjecture needs further exploration for verification.

The combined analysis of m^6^A-seq and RNA-seq data showed that there was a positive correlation between mRNA expression fold change and m^6^A modification abundance; the greater the change in gene expression level, the greater the change in m^6^A peaks’ abundance. This result was the same as the expression relationship between m^6^A and mRNA in bovine myoblasts and yak adipocytes [44,48], but was opposite to the expression relationship characteristics in goose embryonic muscle development [61]. This suggested that the association between mRNA m^6^A abundance and gene expression level was relatively conserved, but may be different in different species, tissues or cells. Further analysis found that there were more “hypo-up” and “hypo-down” genes than “hyper-up” and “hyper-down” genes in the *Longissimus dorsi* muscle of cattle-yaks, which was different from the results of chicken [68], goose [61] and cattle [44]. This phenomenon indicated that genes with reduced methylation levels may be instrumental in muscle growth of cattle-yak. Moreover, compared with the study of cattle-yak testis [47], our results were also unlike, indicating that even in the same species, the types of m^6^A-modifying genes that work in different tissues may be diverse. GO and KEGG analysis were performed to identify the potential roles of differentially methylated genes in muscle development of cattle-yak. GO analysis showed that DMPs and DEGs were mainly enriched in some muscle-related and metabolism-related terms, such as myofibril term, actin filament binding and glycolytic process, and many differentially methylated genes were also involved in the transcriptional regulation of various transcription factors through RNA polymerase. Among them, some members of the *ZNF* family, as key eukaryotic transcription factors, were thought to play an important role in muscle development [69] and were significantly enriched in some RNA transport and skeletal muscle-related terms. According to the KEGG pathway analysis, many classical signaling pathways related to skeletal muscle development were significantly enriched, including Wnt signaling pathway and Hippo signaling pathway. In addition, DMPs and DEGs were also obviously enriched in some fat deposition-related pathways (fatty acid metabolism and PPAR signaling pathways) and plateau adaptation-related classical pathways (HIF-1 signaling pathway). We also discovered that the marker genes that related muscle development were involved in different biological processes, which also testified the potential role of functional genes in the growth and development of *Longissimus dorsi* muscle of cattle-yak. In summary, m^6^A methylation may act as a key part to promote *Longissimus dorsi* muscle development through the classical muscle-related signaling pathways.

In addition to the ubiquitous m^6^A modifications on mRNAs, m^6^A modifications on lncRNAs have also attracted more and more attention from researchers. Recent studies have revealed the promotion of m^6^A modification on lncRNA in the muscle development and muscle fiber type conversion [14,39,70,71], and some researchers reported the dynamic changes of m^6^A-methylated lncRNAs during skeletal muscle formation in mouse C2C12 myoblast cell line [40]. However, there was still no report about the m^6^A methylation characteristics of lncRNAs in the muscle development stage of cattle-yak. Thus, we reassembled the transcripts of all samples using MeRIP-seq and RNA-seq data and stream neural network algorithms in this study. Our study was the first evidence to identify the m^6^A-methylated lncRNAs dynamic alteration during *Longissimus dorsi* muscle development in cattle-yak, which results were consistent with the results of m^6^A-lncRNA in mice [40] and pigs [39]. Moreover, gene expression levels can be regulated by m^6^A modification, for example, *METTL14* knockdown can enhance the expression level of *XIST* through decreasing the m^6^A modification of *XIST*, and m^6^A-methylated *XIST* can also be recognized by *YTHDF2* to mediate *XIST* degradation [72]. Similarly, *METTL3*-mediated m^6^A methylation modification can up-regulate LINC00958, resulting in low overall survival in patients with hepatocellular carcinoma [73]. In our comprehensive analysis, there was a negative correlation between expression levels of lncRNAs and their m^6^A abundance, indicating that m^6^A modifications may negatively regulate the expression of these lncRNAs. Combined with the results of the above methylation enzymes, we found that the expression level of m^6^A reader protein *YTHDF2* was extremely significantly decreased during the development of cattle-yak, and *YTHDF2* has been proved to mediate RNA decay and enhance RNA stability [74,75,76]. Accordingly, it was reasonable that we speculated that *YTHDF2* mainly exerted its effects on identifying and binding m^6^A-modified lncRNAs in the muscle growth of cattle-yaks, thereby regulating the expression level of lncRNAs and making it play a role in promoting the muscle development. It was found by Ponjavic et al. that the co-expression of ncRNAs and adjacent protein-coding genes was a common phenomenon [77]. We analyzed mRNAs near the DMPs lncRNAs; obviously, we found that these genes were closely related to muscle tissue development. *FZD6*, as the target gene of TCONS_00019792, is a receptor for *Wnt* gene and is associated with tissue regeneration [78]. Moreover, the nearby gene *ZNF704* of TCONS_00019740 was bound up with production performance. It was found that the expression abundance of *ZNF704* gene was significantly correlated with pork quality traits using genome-wide association and eQTL analysis [79]. Furthermore, Tao et al. revealed that *ZNF704* was involved in muscle differentiation and metabolism in sheep, in the meantime, it was identified as a candidate gene related to the body weight [80]. Interestingly, the expression levels of these two lncRNAs decreased with the reduction of m^6^A abundance, indicating that m^6^A-enriched lncRNAs may play a crucial role in the early growth and development of cattle-yaks, but the functional mechanism of m^6^A-modified lncRNAs in skeletal muscle development needs further exploration. In addition, there was a noteworthy phenomenon that the DEL targets were not only significantly enriched in muscle-related pathways, but also significantly enriched in hypertrophic cardiomyopathy pathways that was not enriched in previous studies of DNA methylation on yaks [81,82]. Long-term living in high altitude areas may cause myocardial hypertrophy and other high-altitude sicknesses. We speculated that although the cattle-yak has a better ability to adapt to high altitude environment than does cattle, cattle-yak may still have myocardial hypertrophy compared with yak. This may be caused by the lack of abundant elastic fibers in the blood vessels of the cattle-yak heart, which still needs further exploration.

To sum up, this comprehensive work revealed for the first time the transcriptome-wide m^6^A modification and distribution patterns that affected the development of the *Longissimus dorsi* muscle of cattle-yak. We not only described the mRNA m^6^A modification profile, but also explored the lncRNA m^6^A modification landscape. Additionally, we identified the correlation between m^6^A methylation and gene or lncRNA expression levels, and analyzed the potential functions of differentially methylated genes and lncRNAs, indicating the potential regulatory mechanism of m^6^A modifications in skeletal muscle development. This study will provide new insights into the key regulatory role of m^6^A methylation in skeletal muscle development and open up new perspectives for genetic breeding in bovines.

## 4. Materials and Methods

### 4.1. Animal Welfare

All the animal experiments and procedures in this work were implemented strictly in accordance with the regulations of Lanzhou Institute of Husbandry and Pharmaceutical Sciences of the Chinese Academy of Agricultural Sciences (LIHPS, CAAS), and the grant number is LIHPS-20220144. All the animal samples were collected in Gannan Tibetan Autonomous Prefecture, Gansu, China.

### 4.2. Samples Collection and RNA Extraction

The experimental cattle-yaks were raised under the same feed conditions. Three cattle-yaks (6 months old) and three cattle-yaks aged 18-month-old were selected to be slaughtered for the *Longissimus dorsi* muscle. All the samples were stored in liquid nitrogen (−80 °C) for the subsequent RNA isolation.

According to the manufacturer’s instructions, the TRIzol (Invitrogen, Carlsbad, CA, USA) was used to isolate the total RNA. The Thermo Scientific NanoDrop 2000c (ThermoFisher Scientific Inc., Waltham, MA, USA) and 1.5% agarose gel electrophoresis were performed to assess the quality and concentration of the total RNA. Furthermore, the qualified RNA was reversed transcribed into cDNA for the quantitative real-time PCR (qRT-PCR) analysis by the Transcriptor First Strand cDNA Synthesis Kit (Takara Bio lnc., Dalian, China). The RNA and cDNA samples were frozen subsequently at −80 °C.

### 4.3. qRT-PCR and m^6^A RNA Methylation Quantification

The *GAPDH* gene was selected as the reference gene to evaluate the levels of RNA methylation-related genes (*METTL3/14*, *WTAP*, *FTO*, *ALKBH5*, *YTHDF1/2/3*, etc.) by the LightCycler^®^ 96 Instrument (Roche, Basel, Switzerland). All the primer sequences were listed in Table S1. The experimental cycling protocol contained 95 °C (30s) of one cycle for preincubation, 95 °C (5s) and 55 °C (30s) of 39 cycles. Then the protocol stopped at 95 °C (5s). Finally, the melting curve was generated by means of cooling the productions to 65 °C and then heating to 95 °C gradually. All the experiments were run for three times, and the relative gene expression level was calculated using the 2^−ΔΔCt^ method [83]. 

To further measure the global m^6^A levels in the yak skeletal muscle, the EpiQuik RNA Methylation Quantitative Kit (Eigentek, P-9005, Farmingdale, NY, USA) was used to construct the m^6^A standard curve following the manufacturer’s protocol. And absorbance was detected at 450 nm to calculate the global m^6^A levels using a microplate reader (Thermo Scientific, Shanghai, China).

### 4.4. MeRIP-Seq and mRNA Sequencing

The qualified RNA (more than 100 μg and RIN > 7.0) was retained for sequencing in the next step. Then the poly (A) RNA solution was fragmented at 94 °C for 5 min using the thermocycler. Subsequently, the pre-equilibrated m^6^A-Dynabeads was added into the fragmented RNA at room temperature while rotating (tail-over-head) at 7 rotations per minute for one hour. After washing of m^6^A-Dynabeads, elution of m^6^A-positive RNA and extraction and cleanup step of the RIP, a RIP library was constructed using 100 ng of RNA (100 ng of input and 100 ng of post m^6^A-IP positive fraction) utilizing the Illumina TrueSeq Stranded mRNA platform. Finally, Illumina HiSeq X10 system (OE Biotech Co., Ltd., Shanghai, China) was performed to conduct the 2×150 bp paired-end sequencing. The raw data in this work was deposited at the GENE EXPRESSION OMNIBUS (GEO) database (accession number: PRJNA879097).

### 4.5. Data Processing

Raw data underwent a series of processing using the Trimmomatic software with default parameter (sliding window filtering with length 4 and removal of sliding window with average base mass below 15) [84], including removing reads containing adapter, reads containing poly-N and low quality, then the clean data were obtained. Subsequently, the SortMeRNA [85] software was performed to remove ribosomal RNA reads, and the remaining clean reads were mapped to the reference genome (BosGru3.0) using HISAT2 [86] software with default parameters. The high quality of MeRIP-seq data were accessed by the Guitar R package [87] and deeptools [88].

MeTDiff peak calling software was used to identify the m^6^A-enriched peaks in each sample with the corresponding input sample serving as control [89]. The setting conditions for peak detection were FRAGMENT_LENGTH = 200, PEAK_CUTOFF_PVALUE = 0.01 and PEAK_CUTOFF_FDR = 0.05. The peaks collected were annotated using ChIPseeker software [90] by intersection with gene architecture, and the results were visualized by IGV software [91]. After which, the differential peaks of cattle-yaks of different stages were detected by MeTDiff with parameter (‘PEAK_CUTOFF_FDR = 0.05′) and were annotated by ChIPseeker.

The fragments per kilobase of transcript per million reads mapped (FPKM) value were used to calculate the expression levels of the mRNAs and lncRNAs [92]. We performed the DESeq software to screen the differential expression genes (DEGs) and lncRNAs (DELs) according to the criterions of |log2FC| > 1.5 and *p* < 0.05 [93]. The obtained DEGs and DELs were analyzed by GO (http://www.geneontology.org) and KEGG (https://www.genome.jp/kegg/) analysis to further explore the potential functions. The pathways with *p*-value lower than 0.05 was considered significant.

### 4.6. Identification of m^6^A-Modified LncRNAs

The String Tie software was used to reassemble transcripts of all samples [94], then the sample input data was considered as transcriptome data to conduct the lncRNA prediction and analysis combined with known lncRNA annotation information in NCBI and Ensembl databases. The screening and identification of lncRNAs underwent a series of strict steps, as in our previous report [1]. Finally, the detected peak position information was compared with the known lncRNAs annotation information in the NCBI and Ensembl databases. If there was overlap, it was considered that the peak may be on the lncRNA.

All identified peaks on lncRNAs were used to analyze the specific and common peaks of the two groups. Then, the potential lncRNA candidate genes were obtained after removing the transcripts with coding potential. FEELnc software was used to count lncRNA types by the positional relationship between lncRNA and known protein-coding transcripts. The DELs with significant difference in m^6^A peaks were divided into four groups (hyper-methylation and up-regulated group, hyper-methylation and down-regulated group, hypo-methylation and up-regulated group and hypo-methylation and downregulate group). A four-quadrant diagram was generated using the ggplot2 package

## Figures and Tables

**Table 1:** Distribution of lncRNAs with a marked change in both RNA expressions and m^6^A methylation levels between group CY6 and CY18.

Chr	LncRNA	DEP.lg.*P*	DEP log2FC	exp_FC	exp_log2FC	exp_*P* val	Types
1	TCONS_00000157	−2.76	−1.11	0.1356	−2.8819	0.0183	Hypo-down
1	TCONS_00000556	−1.31	−0.88	0.2697	−1.8905	0.0043	Hypo-down
10	TCONS_00002918	−2.10	−1.01	0.5257	−0.9276	0.0444	Hypo-down
18	TCONS_00019792	−1.83	−0.68	0.4036	−1.3089	0.0379	Hypo-down
10	TCONS_00002929	−4.08	−0.66	3.5254	1.8178	0.0080	Hypo-up
29	TCONS_00043230	−1.89	−0.99	0.1666	−2.5855	0.0032	Hypo-down
18	TCONS_00019740	−2.32	−0.92	0.2736	−1.8693	0.0426	Hypo-down
X	TCONS_00065573	−2.12	−0.73	17.9715	4.1676	<0.0001	Hypo-up

## Data Availability

All data generated or analyzed during this study can be found below: https://www.ncbi.nlm.nih.gov/bioproject/PRJNA879097. The accession number is PRJNA879097.

## Supplementary Materials

The following are available online at https://www.mdpi.com/article/10.3390/cells11223654/s1, Figure S1: The heatmap shows DEGs in each sample, Table S1: The details of all the primer sequences in this study, Table S2: Summary of Sequencing Reads and Mapping to the Reference Genome, Table S3: The details of DMPs, Table S4: The details of DEGs between two groups, Table S5: The identified genes with four types, Table S6: The details of DELs between two groups.
