# Supplementary figures and images for: Transcriptome-Wide Study of mRNAs and lncRNAs Modified by m6A RNA Methylation in the Longissimus Dorsi Muscle Development of Cattle-Yak
Source: Cells. 2022 Nov 17;11(22):3654. doi: 10.3390/cells11223654 (PMC9688506; doi:10.3390/cells11223654)

CY18-vs-CY6:p-value<0.05&& |log2FC|>0.58

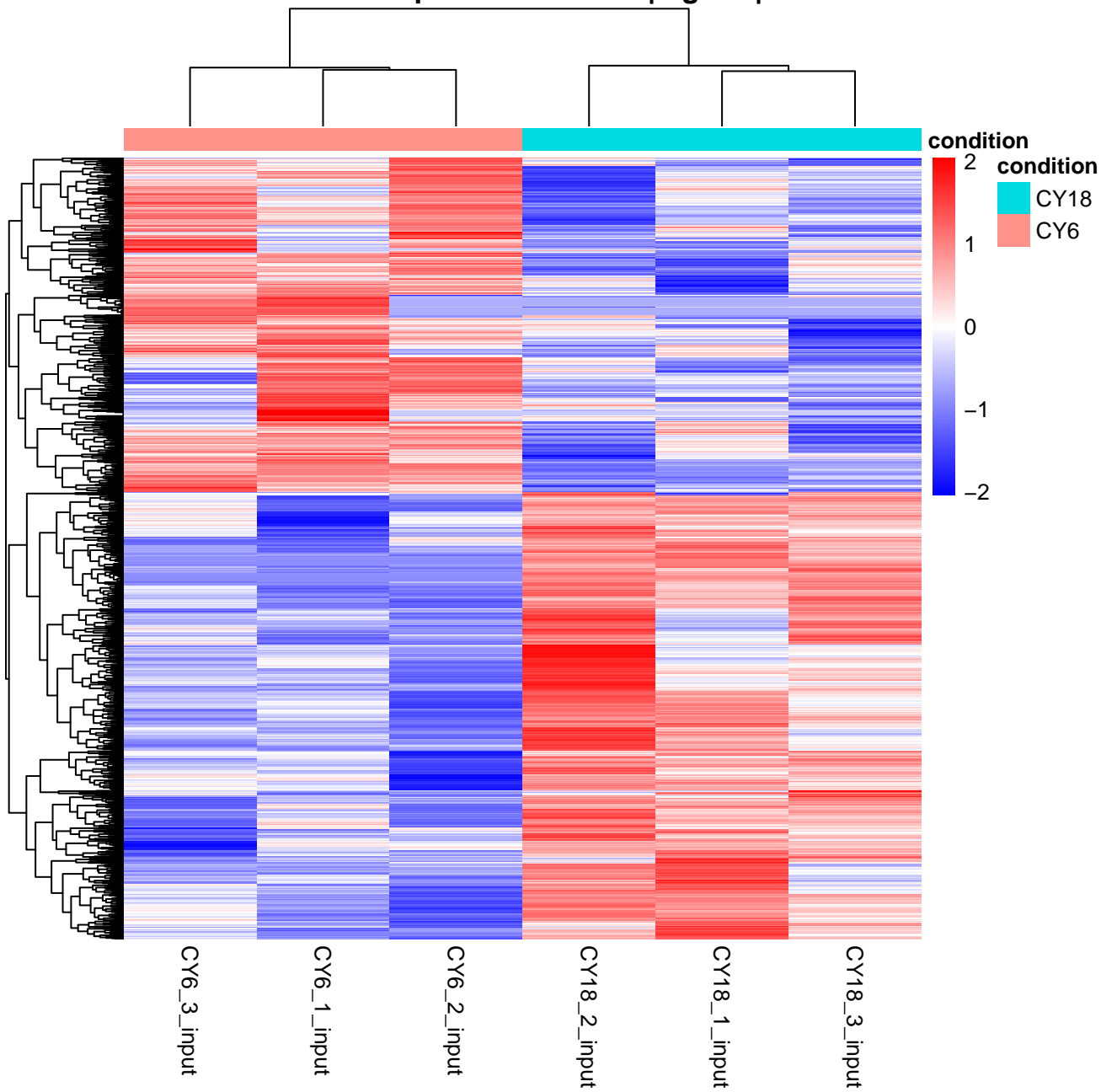

Supplement: Supplementary file 1 [file cells-11-03654-s001.zip › Supplementary Figure S1.pdf]
